# *Salmonella enterica* Serovar Enteritidis, Japan

**DOI:** 10.3201/eid0912.030172

**Published:** 2003-12

**Authors:** Hidemasa Izumiya, Naomi Nojiri, Yoshiko Hashiwata, Kazumichi Tamura, Jun Terajima, Haruo Watanabe

**Affiliations:** * National Institute of Infectious Diseases, Tokyo; † Hiroshima City Institute of Public Health, Hiroshima, Japan

**Keywords:** *Salmonella* Enteritidis, R-plasmid, ampicillin resistance, phange type conversion

**To the Editor**: Nontyphoidal salmonellae are the important causative agents of foodborne diseases in Japan and other industrialized countries. *Salmonella enterica* serovar Enteritidis has risen to the leading cause of infection among *Salmonella* spp. since 1989 (*1*). Emergence of drug-resistant *S*. Enteritidis has been rarely reported while *S*. Typhimurium, another serovar of major public health concerns, has been reported to acquire multidrug resistance such as DT104 resistance to ampicillin, chloramphenicol, streptomycin, sulfonamide, and tetracycline (R-ACSSuT) (*2*).

We previously reported outbreaks caused by strains resistant to ampicillin and streptomycin (resistance type R-AS, herein); the strains’ reactions against the phages used in bacteriophage typing did not conform to any known reaction patterns (phage type [PT] RDNC-a, herein, with the following reactions: (-) for 3, 5–7, 11–13, 15, and 16 phages; (+++) for #2 phage; opaque lysis [OL] for #4 and #9 phages; <OL for #10 phage; and ambiguous reactions (-/+++) were observed for 1, 8, and 14 phages) (*3*). To investigate the characters of the R-AS strains more extensively, we surveyed isolates from outbreaks that occurred from 1997 to 2002 for antimicrobial drug susceptibility and bacteriophage typing.

*S*. Enteritidis strains from 899 outbreaks that occurred from 1997 to 2002 were tested. Bacteriophage typing was done according to the Public Health Laboratory Service (PHLS), London, United Kingdom guidelines (*4*). Antimicrobial drug susceptibility testing was done with a disc diffusion method on Mueller-Hinton II agar (Becton Dickinson Microbiology Systems, Cockeysville, MD) as previously described (*5*). Antimicrobial drugs used in this study were ampicillin, streptomycin, tetracycline, kanamycin, nalidixic acid, gentamycin, sulfamethoxazole-trimethoprim, trimethoprim, chloramphenicol, cefotaxim, and ciprofloxacin.

Dominant phage types were PT4 (36.9%) and PT1 (26.9%). They have been dominant among outbreak-related strains since 1992 (*1*). Other types were also identified at certain frequencies. For example, RDNC-a, PT47, PT6, PT6a, and PT21 accounted for 4.4%, 5.3%, 4.0%, 3.2%, and 2.0% of the phage types, respectively.

Strains sensitive to all the antimicrobial drugs tested were the most predominant (55.1%), followed by those resistant to only streptomycin (34.8%). R-AS was the third most predominant, accounting for 4.1%. A correlation existed between drug resistance and phage types in that all the R-AS strains (n = 37) showed RDNC-a in bacteriophage typing, and all the RDNC-a strains (n = 40) were resistant to at least ampicillin including two R-A and one R-AST strains.

Since previous studies described the correlation between drug resistance and phage types as a result of acquisition of an R-plasmid (*6*), we focused on the relationship between RDNC-a and ampicillin resistance. Plasmid profiles analysis of the RDNC-a strains showed that all but one (R-AST) had at least two kinds of plasmids, and all but one were approximately 50 kb and 60 kb in size. The last could be the so-called serovar-specific plasmid (*7*). Southern blot analysis by using the ampicillin resistance gene of pBluescript KS (+) (Stratagene, La Jolla, CA) as a probe indicated that a resistance gene was carried on the 50-kb plasmid. Furthermore, when *Escherichia coli* DH10B cells (Invitrogen Corporation, Carlsbad, CA) were transformed with plasmids isolated from an RDNC-a R-AS strain and plated onto Luria broth plates containing 100 mg/L of ampicillin, the 50-kb, but not 60-kb, plasmid could be isolated from the ampicillin-resistant transformants. And the 50-kb plasmid from the transformants was hybridized to the probe for ampicillin resistance described above. Thus, the 50-kb plasmid of RDNC-a R-A or -AS strains was suggested to be an R-plasmid responsible for ampicillin resistance.

A representative 50-kb plasmid (p981123) was prepared from the DH10B transformant cells described earlier for further characterization. Southern blot analysis suggested that a 6-kb *Eco*RI fragment contained the resistance determinant. Sequences for the fragment were analyzed done by using ABI PRISM 310 sequencer and BigDye Terminator Cycle Sequencing Ready Reaction Kit (Applied Biosystems, Foster City, CA). The resulting sequence showed high similarities to *Pseudomonas aeruginosa* Tn801 (accession no. AF080442; 98% identical) and *E. coli* Tn3 (accession no. ISTN3X; 96% identical), comprising one of Tn3-like inverted repeats and putative coding regions for transposase, resolvase (also called repressor), and ampicillin resistance. The resistance gene encodes a TEM-1 type β-lactamase. (The sequence has been registered to DDBJ/GenBank/EMBL with accession no. AB103092.)

Conjugative transferability of p981123 between *S*. Enteritidis strains was examined by using the parental *S*. Enteritidis RDNC-a R-AS strain as a donor, and three independent *S*. Enteritidis strains (PT1; PT4; and PT21) resistant to nalidixic acid (R-N) as recipients. p981123 was transferable between *S*. Enteritidis strains at frequencies of 10^-5^ to 10^-4^, and the resulting R-AN transconjugant showed the same lytic pattern of the typing phages as RDNC-a. Thus, transfer of p981123 could convert the phage types at least from PT1, PT4, and PT21 to RDNC-a. Pulsed-field gel electrophoresis (PFGE) was done by using *Xba*I or *Bln*I as well, and RDNC-a strains showed a variety of PFGE profiles. These results suggest emergence and prevalence of the 50-kb R-plasmid converting phage types to RDNC-a in *S*. Enteritidis in Japan.

Previous studies reported correlation between R-plasmids and phage types of *S*. Enteritidis, where, for example, a 34-MDa R-plasmid of incompatibility group N (IncN) (*8*) and a 36-MDa R-plasmid of IncX (pDEP57) (*6*) were described. Both kinds of plasmids encoded ampicillin resistance as well as that in this study, but both were identified in PT6a isolates. Preliminary sequence data of the region of p981123 essential for replication indicated a gene coding for a protein similar to protein p1 of R6K (IncX) plasmid (*9*), which suggests that p981123 may be related to pDEP57. However, the reactions to the typing phages in RDNC-a strains were different from those in PT6a. Therefore, the R-plasmid in this study seems to have different features from previous ones. In addition, *S*. Enteritidis PT6d resistant to ampicillin was recently reported (*10*). Relationship between RDNC-a in this study and PT6d is unknown, and further investigations will be needed.

Transfer of an R-plasmid is a common way for bacteria to acquire drug resistance, and it often affects other aspects such as sensitivity of bacteriophages, as described in this study. Molecular based surveillance for drug resistance in *S*. Enteritidis needs to continue.
